# Roles of Osteopontin Gene Polymorphism (rs1126616), Osteopontin Levels in Urine and Serum, and the Risk of Urolithiasis: A Meta-Analysis

**DOI:** 10.1155/2015/315043

**Published:** 2015-02-15

**Authors:** Xiao Li, Kang Liu, Yongsheng Pan, Jing Zhang, Qiang Lv, Lixin Hua, Zengjun Wang, Jie Li, Changjun Yin

**Affiliations:** Department of Urology, The First Affiliated Hospital of Nanjing Medical University, Nanjing 210029, China

## Abstract

*Objective*. Previous studies have investigated the relationships between osteopontin gene polymorphism rs1126616 and OPN levels and urolithiasis, but the results were controversial. Our study aimed to clarify such relationships. *Methods*. A meta-analysis was performed by searching the databases Pubmed, Embase, and Web of Science for relevant studies. Crude odds ratios (ORs) or standardised mean differences with 95% confidence intervals (CIs) were calculated to evaluate the strength of association. Publication bias was estimated using Begg's funnel plots and Egger's regression test. *Results*. Overall, a significantly increased risk of urolithiasis was associated with OPN gene polymorphism rs1126616 for all the genetic models except recessive model. When stratified by ethnicity, the results were significant only in Turkish populations. For OPN level association, a low OPN level was detected in the urine of urolithiasis patients in large sample size subgroup. Results also indicated that urolithiasis patients have lower OPN level in serum than normal controls. *Conclusion*. This meta-analysis revealed that the T allele of OPN gene polymorphism increased susceptibility to urolithiasis. Moreover, significantly lower OPN levels were detected in urine and serum of urolithiasis patients than normal controls, thereby indicating that OPN has important functions in the progression of urolithiasis.

## 1. Introduction

Urolithiasis is one of the most common diseases that affect all ethnicities and populations worldwide. Urinary stone formation is a complicated disorder involved in a process of nucleation, growth, aggregation, and retention of crystals in the urine [1, 2]. Among all types of urinary stones, calcium, struvite, uric acid, and cystine stones account for 70% to 80%, 5% to 10%, 5% to 10%, and 1%, respectively [3]. In recent years, despite considerable efforts by researchers, the lithogenic and inhibitory pathogenic mechanisms of urolithiasis remain unknown.

Urolithiasis is associated with acidic macromolecules, which direct nucleation, growth, and morphology by both molecular templating and preferential adsorption onto specific crystal faces [4, 5]. Osteopontin (OPN) is a unique acidic macromolecule with the crucial biological function of inhibiting urinary crystallization by possibly reducing the growth and aggregation of calcium oxalate (CaO_X_) crystals and inhibiting the binding of CaO_X_ crystals directly to renal tubular epithelial cells [6, 7]. Nonetheless, osteopontin is presumably one of the most important components in the calcium stone matrix, implying its potential role in urolithiasis.

As a multifactorial disease, urolithiasis is associated with the effects of multiple genes combined with lifestyle and environmental influences [8]. Familial clustering of idiopathic urolithiasis has been confirmed by a growing number of epidemiologic studies, suggesting that genetic factors play a significant role in urinary stone formation [9]. Osteopontin is a 44 kDa negatively charged acidic hydrophilic multifunctional protein that contains a functional Gly-Arg-Gly-Asp-Ser cell-binding sequence and encoded by the OPN gene, which is located on chromosome 4q21-25 and spans approximately 11 kb with 7 exons [10–13]. The single nucleotide polymorphism rs11226616 of OPN gene means +750 C > T in exon VII of 3′ UTR, which can also be expressed by OPN C6982T polymorphism. Genetic variations of the OPN gene have been described, and some of these variations were reportedly associated with calcium in patients with urolithiasis [14–16], showing that rs1126616 can potentially explain urolithiasis susceptibility. However, whether rs1126616 of OPN gene can influence urolithiasis remains unclear.

Though previous studies showed the associations between OPN gene polymorphism (rs1126616) and OPN levels with urolithiasis, the information in each of the published studies was limited, and the results were inconsistent or even contradictory. Thus, we performed a meta-analysis of all eligible studies to provide a more accurate estimate of the association of the OPN gene polymorphisms and the risk of urolithiasis. Additionally, the differences in urine and serum OPN levels between patients with urolithiasis and the control subjects were also compared.

## 2. Materials and Methods

We conducted a comprehensive search of PubMed, Embase, and Web of Science for relevant studies on the association between OPN gene polymorphism (rs1126616) and OPN levels with urolithiasis. Our search covered all the papers published up to 30 September 2014. The combinations of the following keywords were used: “Osteopontin” or “OPN,” “polymorphism” or “level,” and “Urolithiasis” or “Urinary calculi” or “Urinary stone.” We collected additional literature by manual searching of the references of original studies or reviews. Only the latest or most comprehensive sample size was included if studies had partly overlapping subjects. If the same case series were used in more than one article, only the study with the largest sample size was selected.

Studies involved were selected if they met the inclusion criteria as follows: (1) case-control design was used; (2) for genotype frequency association, the number of each genotype must be clear and sufficient data must be provided for calculating the odds ratio (OR) and the corresponding 95% confidence interval (CI); (3) for OPN levels association, the study must have clear original data on the OPN levels in urine or serum. The major exclusion criterion was duplicates of previous publication.

### 2.1. Data Extraction

Two investigators (Xiao Li and Kang Liu) independently and carefully reviewed the identified studies to determine whether an individual study was eligible for inclusion. The investigators independently extracted data from studies involved, and disagreements were resolved by discussion. The following data were collected from each study: first author's name, year of publication, ethnicity, genotyping method, source of controls, number of cases and controls, frequency of rs1126616 gene polymorphism in cases and controls, the mean of OPN levels, and SD. All data were recorded in a standardised form.

### 2.2. Statistical Analysis

The pooled ORs or standardised mean differences (SMD) with 95% CI were used to, respectively, evaluate the strength of association between the rs1126616 genotype and OPN levels with urolithiasis susceptibility. We used SMD because the OPN levels were measured by different methods in the included studies. A 95% CI without 1 for OR indicated a significantly increased or reduced urolithiasis risk. The fixed-effects model (Mantel-Haenszel method) and the random effects model (DerSimonian-Laird method) were used to pool the data [17]. Sensitivity analysis involved calculating the results again by omitting one single study each time. Metaregression analysis was performed to explore the sources of heterogeneity if significant heterogeneity was present among studies. Subgroup analysis was performed by ethnicity and genotyping methods. Begg's funnel plots and Egger's linear regression test were used to examine the publication bias between the studies, and a *P* < 0.05 was considered significant [18]. Hardy-Weinberg equilibrium (HWE) was evaluated using the goodness-of-fit chi-square test, and *P* < 0.05 was regarded a significantly selective bias [19].

All statistical analyses were performed with Stata software (version 12.0; StataCorp LP, College Station, TX). *P* values were all two-sided and were considered statistically significant when being less than 0.05.

## 3. Results

### 3.1. Studies Characteristics

**Figure 1:**
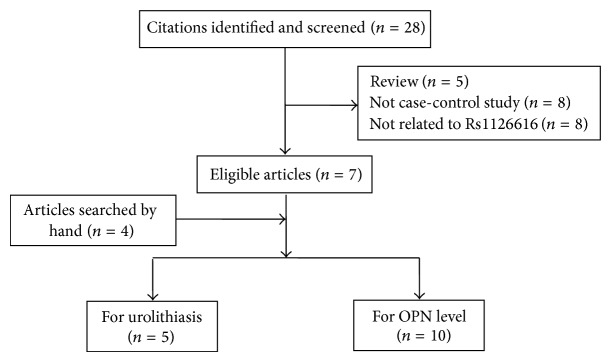
Flow diagram of literature search and selection process.

A total of 11 case-control studies were involved in the meta-analysis [14, 16, 20–28], and the details of the studies containing data on OPN gene polymorphism (rs1126616) and OPN levels in serum or urine are shown in Tables 1 and 2, respectively.  Figure 1 shows the flowchart of the literature search and selection process. All the source of controls was the healthy population. DNA was extracted from whole blood in all these studies, and two genotyping methods were used, namely, polymerase chain reaction- (PCR-) restriction fragment length polymorphism (RFLP) and PCR-single-strand conformation polymorphism (SSCP). Urolithiasis was confirmed by ultrasonography or radiography in all studies.

### 3.2. Meta-Analysis Results

**Figure 2:**
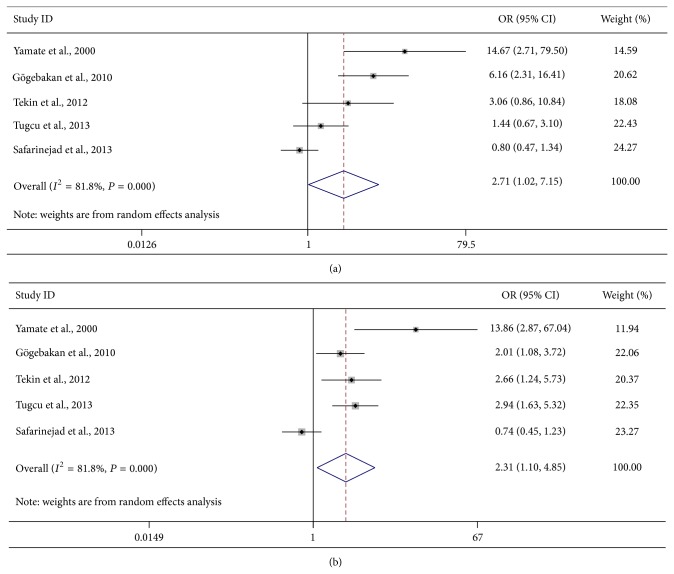
Forest plots of urolithiasis associated with distribution of genotypic frequencies of rs1126616. (a) Homozygote model; (b) dominant model.

**Figure 3:**
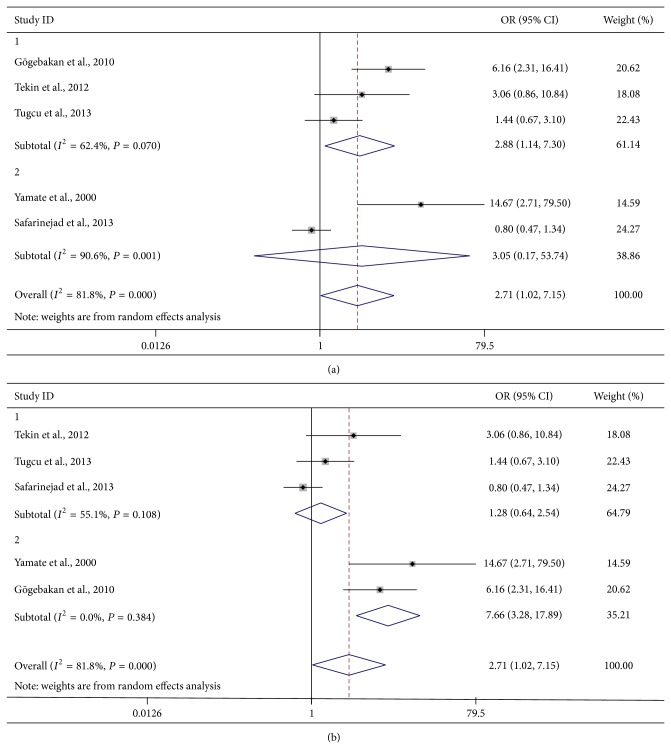
Forest plots of subgroup analysis of urolithiasis associated with the distribution of genotypic frequencies of rs1126616 in the homozygote model: (a) stratified by ethnicity; (b) stratified by genotyping method.

The main results of the meta-analysis of the association between OPN gene polymorphism rs1126616 and urolithiasis are listed in Table 3. Overall, the pooled OR was 2.49 (95% CI: 1.01–6.17) for heterozygote model, 2.71 (95% CI: 1.02–7.15) for homozygote model, 2.31 (95% CI: 1.10–4.85) for dominant model, and 1.64 (95% CI: 0.95–2.81) for recessive model (Figure 2). When the studies were stratified by ethnicity, the results were positive only in Turkish populations (heterozygote model: pooled OR = 2.69, 95% CI: 1.26–5.75; homozygote model: pooled OR = 2.88, 95% CI: 1.14–7.30; dominant model: pooled OR = 2.50, 95% CI: 1.72–3.63; Figure 3(a)). Moreover, when the studies were stratified by genotyping method, the result was significant only in the PCR-SSCP method, with a pooled OR of 7.66 (95% CI: 3.28–17.89) for the homozygote model and 2.86 (95% CI: 1.12–7.33) for the recessive model (Figure 3(b)).

**Figure 4:**
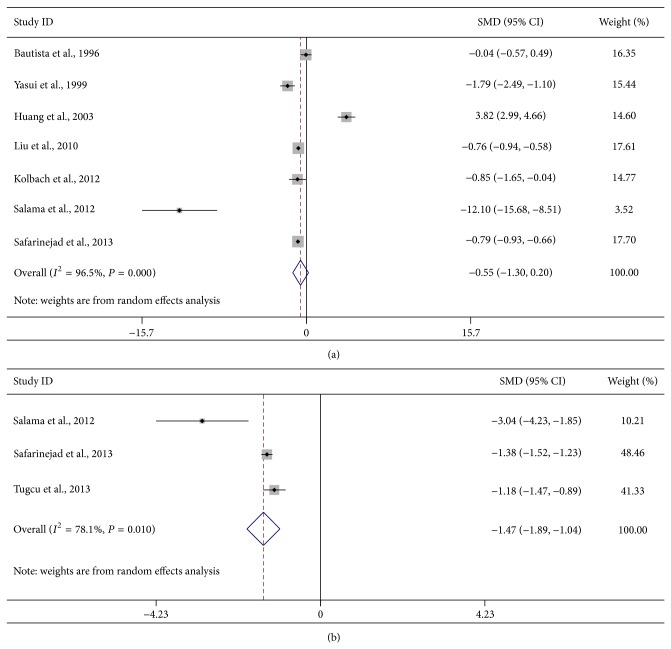
Forest plots of urolithiasis associated with OPN levels. (a) OPN levels in urine; (b) OPN levels in serum.

For the association of OPN level, the detailed results are shown in Table 4. The pooled SMD was −0.55 (95% CI: −1.30–0.20) for the association between OPN levels in urine and urolithiasis risk (Figure 4(a)). When the studies on OPN level in urine were stratified by ethnicity, the results were negative in both the Asian and Turkish subgroups with a pooled SMD of −1.49 (95% CI: −3.79–0.81) and −0.55 (95% CI: −1.30–0.20). However, the results were positive when the studies were stratified by sample size, and a low OPN level was found in urine of urolithiasis patients in large sample size subgroup. Furthermore, our results indicated that reduced OPN level was evident in the serum of urolithiasis patients compared with normal controls (SMD = −1.47, 95% CI: −1.89 to −1.04; Figure 4(b)).

### 3.3. Test of Heterogeneity

For OPN gene polymorphism rs1126616 association, a significant heterogeneity was observed in all the genetic models. However, heterogeneity decreased when subgroup analyses were conducted by ethnicity or by using genotyping methods. For the OPN levels association in urine or serum, heterogeneity between studies was also observed in overall comparisons, as well as in subgroup analyses. However, heterogeneity was also reduced by subgroup analyses. We failed to confirm the source of the heterogeneity because of overmuch confounding factors.

### 3.4. Sensitivity Analysis

**Figure 5:**
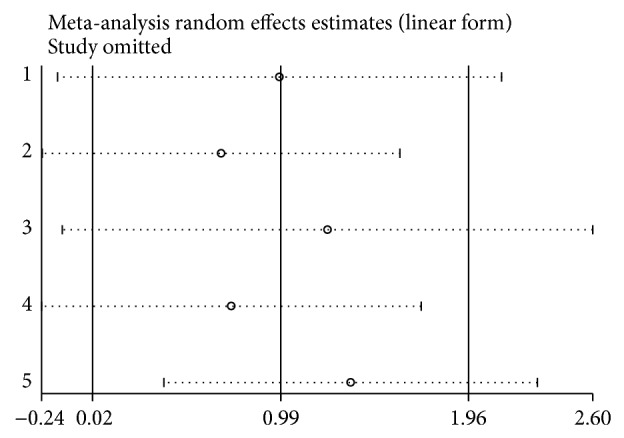
Sensitivity analysis of urolithiasis risk associated with the OPN polymorphism under the homozygote model.

Sensitivity analysis was used to detect the influence of each study on the pooled OR by repeating the meta-analysis while omitting a single study each time [29].  Figure 5 shows the sensitivity analyses for OPN gene polymorphism association for the homozygote model in the overall population, thereby demonstrating that no individual study significantly affected the pooled OR. The sensitivity analysis indicated that our results were reliable.

### 3.5. Publication Bias

**Figure 6:**
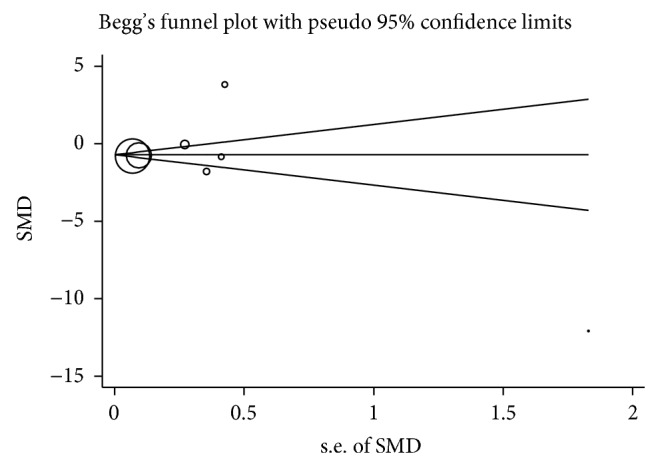
Begg's funnel plot for publication bias test of urolithiasis risk associated with the urine OPN levels.

Begg's funnel plot was utilised to assess the publication bias of the literature.  Figure 6 displays a funnel plot for the urine OPN levels association, thereby indicating the absence of significant publication bias, which was confirmed by Egger's test (*P* = 0.854). Moreover, no significant publication bias was detected for serum OPN levels by Egger's test (*P* = 0.563). For the OPN gene polymorphism association, no significant publication bias was detected as well (dominant model: Begg's test: *P* = 0.221; Egger's test *P* = 0.052).

## 4. Discussion

Urolithiasis, as a complex and multifactorial disease, is one of the most prevalent disorders in the urological system with increasing incidence. Approximately 5% of females and 12% of males are likely to develop urolithiasis during their lifetime [30]. A growing body of evidence shows that organic substances, in addition to inorganic substances, may significantly influence the development of urolithiasis. Osteopontin, a component of the urinary stone matrix, is recognised as a potential protectant against urolithiasis [31]. Thus, mutations in the gene that directs the synthesis of OPN might contribute to urolithiasis formation as a genetic factor. Single nucleotide polymorphisms have been identified as a powerful tool for predicting complex diseases in recent years [32]. However, previous genetic epidemiological studies about the association between OPN gene polymorphism rs1126616 and the risk of calcium urolithiasis are limited, and the results of these studies were inconclusive. For instance, Tugcu et al. advocated that the T allele of OPN gene polymorphism rs1126616 was a risk factor for urolithiasis [25]. Interestingly, Safarinejad and his colleagues held an opposite opinion [26].

Meta-analysis is a powerful tool that can provide more reliable results than a single study and explain controversial conclusions [33]. Thus, we used a meta-analysis to clarify the possible association between the OPN gene polymorphism rs1126616 and the risk of urolithiasis. Moreover, the association between OPN levels and urolithiasis was also evaluated. To the best of our knowledge, no meta-analysis was conducted on this subject before. Finally, for the OPN C6982T polymorphism association, our results indicated that the T allele of C6982T polymorphism might be a risk factor for urolithiasis. When stratified by ethnicity, the results were positive only in Turkish. Moreover, the results were significant only in PCR-SSCP subgroup when stratified by genotyping method. For the OPN level association, a low OPN level was found in the urine of urolithiasis patients in large sample size when stratification was performed according to sample size. Moreover, studies confirmed that OPN level in the serum of urolithiasis patients was lower than normal controls.

Previous studies showed that the occurrence of urolithiasis in particular geographical areas is statistically higher than elsewhere in particular countries [34, 35], suggesting that the incidence of gene polymorphisms can notably vary among different racial populations because of ethnic differences. As a result, stratified analysis was performed by ethnicity in this meta-analysis. Although the exact mechanism was not well known, the results above may be partially explained. First, the sample size was relatively small, which may not reveal the real association because of limited statistical power. Particularly, the research on the Turkish population cannot be generalised to the Caucasian populations. Moreover, different genetic backgrounds and environmental contexts might not be totally reflected by the differences among ethnic groups, as shown by the identical results obtained in Asian and Turkish subgroups. Furthermore, the diversity of variable factors, such as different matching criteria and selection bias, should be taken into consideration.

In recent years, various molecular techniques have been used as tools in genotyping. However, different research methods may affect the conclusions. Therefore, we also conducted a subgroup analysis by using genotyping methods. In this meta-analysis, both PCR-RFLP and PCR-SSCP were applied to analyse the association between OPN gene polymorphism rs1126616 and urolithiasis. However, the subgroup analysis was significant only in PCR-SSCP. As a highly sensitive technique, PCR-SSCP is widely applied in the detection of small sequence changes and point mutations [36]. Nevertheless, diverse methods have advantages in different aspects, which might constitute a source of bias. As a result, the meta-analysis results would be more accurate if the genotyping methods were unified.

Osteopontin is a multifunctional protein expressed in a variety of different cell types in the human body and is synthesised by the kidney and secreted into urine by epithelial cells including those in loops of Henle, distal convoluted tubules, and papillary epithelia [12, 37]. Notably, OPN levels are vital in the mechanism of urolithiasis; therefore, we also evaluated the relationships between OPN levels in urine or serum and urolithiasis. Previous studies suggested that high urinary excretion of osteopontin may play an important protective role in preventing calcium urolithiasis formation, which was further confirmed in our meta-analysis by the lower levels of OPN in both urine (stratified by sample size) and serum of urolithiasis patients than those of normal controls. Possibly, the incorporation of OPN into growing stones might lead to less OPN in patients [38]. However, the accurate mechanism requires further exploration.

Despite the overall robust statistical evidence generated through this analysis, a number of limitations were identified. First, some articles involved in the OPN gene polymorphism association are not in accord with the HWE, which may be attributed to the limited sample size and difference in ethnicity. Meanwhile, significant heterogeneity between studies was observed, and such heterogeneity could be reduced by subgroup analysis. However, as the first meta-analysis regarding the comprehensive assessment of the association, the number of published studies was limited and sample size was relatively small. As a result, the HWE violation and significant heterogeneity were inevitable to some degree. Moreover, our results were based on unadjusted estimates. Consequently, a more precise analysis could be performed in the presence of all individual raw data, which could allow for the adjustment of other covariates, including age, sex, drinking status, and cigarette consumption. Third, urolithiasis results from complex interactions between a variety of genetic and environmental factors, thereby suggesting that urolithiasis susceptibility would not be influenced by any single gene. We cannot exclude the fact that other genetic polymorphisms are linked with rs1126616 or even directly contribute to urolithiasis. Thus, further research with combined effects analysis is required. Last but not least, because of the limited studies involved, the significant difference of results should be interpreted cautiously. Accordingly, more studies should be conducted to provide a more definitive conclusion. To illustrate, the populations in this study only included Asians and Turkish population. Thus, populations of other ethnicities should be involved in future studies.

## 5. Conclusion and Recommendation

Despite these limitations, the results of the present meta-analysis suggest that the carriers of the T allele had higher urolithiasis risk than the noncarriers, especially in individuals of Turkish ethnicity. In addition, lower OPN levels were detected in urine and serum of urolithiasis patients than normal controls, which proved the important role of OPN in the development of urolithiasis. Our research might provide new insight into the aetiology of urolithiasis. The OPN gene polymorphism and OPN level might be an index for detecting the risk of urolithiasis, which could be applied in early screening and prediction of urolithiasis. Nevertheless, several problems require solutions, though we believe that the important roles of OPN gene polymorphism rs1126616 and OPN level are promising. To develop a more comprehensive understanding of the relationship between OPN gene polymorphism and urolithiasis sensibility, the following recommendations should be considered. (1) High-quality studies by standardised unbiased methods are needed, and these studies can offer more detailed individual data. (2) A more comprehensive and generalizable conclusion can be achieved in studies that involve various ethnic groups. (3) Combined effects of different gene polymorphisms need to be analysed. Because the genetic background of stone formation is a complicated issue and includes single-candidate genes and the epigenetic process, a single gene is difficult to be identified as responsible for urolithiasis.

## Figures and Tables

**Table 1:** Characteristics of studies included in the meta-analysis for association between OPN C6982T polymorphism and urolithiasis.

OPN rs1126616							Case (*N*)			Control (*N*)		
Year	Author	Ethnicity	Genotyping	SOC	Case	Control	CC1	CT1	TT1	CC0	CT0	TT0
2013	Tugcu et al. [25]	Turkish	PCR-RFLP	PB	127	92	66	42	19	70	8	14
2013	Safarinejad et al. [26]	Asian	PCR-RFLP	PB	342	684	27	143	172	41	315	328
2012	Tekin et al. [27]	Turkish	PCR-RFLP	PB	64	50	27	27	10	33	13	4
2010	Gögebakan et al. [28]	Turkish	PCR-SSCP	PB	121	100	23	67	31	32	61	7
2000	Yamate et al. [14]	Asian	PCR-SSCP	PB	65	36	2	39	24	11	16	9

SOC: source of controls, PB: population-based study.

**Table 2:** Characteristics of individual studies included in the meta-analysis of OPN level and urolithiasis.

OPN level in urine										
Year	Author	Ethnicity	Country	Sample size	Case	Mean	SD	Control	Mean	SD
2013	Safarinejad et al. [26]	Asian	Iran	1026	342	0.028	0.021	684	0.048	0.027
2012	Salama et al. [24]	Asian	Saudi Arabia	25	15	21.67	3.7	10	58.88	1.7
2012	Kolbach et al. [23]	Caucasian	USA	26	13	2.5	1.5	13	4.0	2
2010	Liu et al. [16]	Asian	China	496	249	0.029	0.024	247	0.050	0.031
2003	Huang et al. [20]	Asian	China	64	32	101.8	13.1	32	55.9	10.8
1999	Yasui et al. [21]	Asian	Japan	60	47	35.77	27.92	13	88.79	35.28
1996	Bautista et al. [22]	Caucasian	England	57	34	0.76	0.71	23	0.79	0.76

OPN level in serum										
Year	Author	Ethnicity	Country	Sample size	Case	Mean	SD	Control	Mean	SD

2013	Safarinejad et al. [26]	Asian	Iran	1026	342	4.2	1.6	684	6.4	1.6
2013	Tugcu et al. [25]	Caucasian	Turkey	219	127	4.5	2.8	92	8.3	3.7
2012	Salama et al. [24]	Asian	Saudi Arabia	25	15	11.20	2.30	10	20.25	3.80

**Table 3:** Meta-analysis results of the association between OPN C6982T polymorphism and urolithiasis risk.

	*N* ^a^	Sample size	CT versus CC	*P* ^b^	TT versus CC	*P* ^b^	CT/TT versus CC	*P* ^b^	TT versus CT/CC	*P* ^b^

Total	5	1681	**2.49 (1.01–6.17)**	0.000	**2.71 (1.02–7.15)**	0.000	**2.31 (1.10–4.85)**	0.000	1.64 (0.95–2.81)	0.024

Ethnicity										
Asian	2	1127	2.75 (0.15–51.89)	0.001	3.05 (0.17–53.74)	0.001	2.91 (0.16–52.75)	0.000	1.14 (0.89–1.46)	0.329
Turkish	3	554	**2.69 (1.26–5.75)**	0.052	**2.88 (1.14–7.30)**	0.070	**2.50 (1.72–3.63)**	0.669	2.08 (0.76–5.67)	0.030

Genotyping										
PCR-RFLP	3	1359	2.07 (0.56–7.67)	0.000	1.28 (0.64–2.54)	0.108	1.76 (0.68–4.57)	0.001	1.11 (0.88–1.42)	0.549
PCR-SSCP	2	322	3.97 (0.47–33.24)	0.014	**7.66 (3.28–17.89)**	0.384	4.58 (0.70–30.18)	0.024	**2.86 (1.12–7.33)**	0.134

^a^Number of studies.

^b^ *P* value of *Q* test for heterogeneity.

**Table 4:** Summary of SMD and 95% CI for associations between OPN level and urolithiasis risk.

Polymorphism	Subgroup	*N* ^a^	Sample size	SMD (95% CI)	*P* value	*P* heterogeneity
Urine	All	7	1754	−0.55 (−1.30–0.20)	0.151	**<**0.0001
	Large sample size	2	1522	−**0.78 (−0.89 to −0.67)**	**<0.0001**	0.749
	Asian populations	5	1671	−0.65 (−1.61–0.31)	0.182	**<**0.0001

Serum	All	3	1270	−**1.47 (−1.89 to −1.04)**	**<0.0001**	0.01

^a^The number of studies.
